# The Veterinarian as a Naturalist1A paper presented before the Louisiana Society of Naturalists, at New Orleans, April 7, 1899.

**Published:** 1899-05

**Authors:** W. H. Dalrymple

**Affiliations:** Baton Rouge, La.


### THE JOURNAL

OF

### COMPARATIVE MEDICINE AND VETERINARY ARCHIVES.

VOL. XX. *MAY, 1899.* No. 5.

### THE VETERINARIAN AS A NATURALIST.[1]

BY W. H. DALRYMPLE, M.R.C.V.S.,
BATON ROUGE, LA.

IT affords me a great deal of pleasure to appear before you this evening in the rôle of essayist. I must confess, however, that when asked by our worthy Secretary to prepare a paper I had some difficulty in deciding upon a subject apropos to the Society and to the occasion. It is true the veterinarian, or I might I rather designate him the student of comparative medicine, is brought daily in contact with nature in many of her phases, and is required to have a sort of conglomerate knowledge concerning her many intricacies; still, when brought face to face with the fact that a paper was expected of me—which I realized would be most acceptable to a society of this nature if along some definite line of work—I was almost about to give up in despair.

In the domain of the naturalist, the field open to the veterinarian is indeed a very wide and varied one; but to attain to any degree of success along any of the many branches of the work he must become a specialist. Many such men are to be found to-day in the ranks of the veterinary profession, some of whom have reached a high degree of eminence; but when we consider the large majority of veterinarians who have not the opportunity or the time to specialize, but who have to keep adding to their store of general knowledge of all subjects pertaining to their professional work, for the purpose of satisfying the demands of a public who expect them to be quite *au fait* in each and every branch of it, the difficulty, or might I rather say the impossiblity (?) of pursuing any special line of study must be apparent. It was just such difficulty that seemed

[1] A paper presented before the Louisiana Society of Naturalists, at New Orleans, April 7, 1899.

to confront me in preparing a paper suitable to be read on such an occasion as this; but there was one thought from which I seemed to obtain a modicum of consolation. There used to be a saying among whist-players to the effect that "Whenever uncertain what to play, always play trumps." Being an ardent devotee of my branch of the healing art, I made the application in this way: If you are undecided as to the choice of a subject, do not lose the opportunity, but say something in behalf of your profession; there are, perhaps, more nature studies embraced by it than by almost any other, and this fact is, as yet, not fully appreciated in our Southern country. Such, then, was a part of the line of argument I advanced to myself and by which I was led to select, as the topic of my paper, "The Veterinarian as a Naturalist," which I hope may be at least interesting if not instructive.

We are told by Hoblyn, in his *Dictionary of Terms Used in Medicine and the Collateral Sciences*, that the naturalist was formerly a denier of revealed truth, of any but natural religion; but now an investigator (and often a devout one) of nature and her laws. Placing the same construction on the term as Hoblyn, the veterinarian ought really to take high rank as a naturalist; and in what follows, in as modest a way as possible, I shall endeavor to give some of the reasons why.

As all of you are aware, anatomy is the foundation study of all medical work; but it may be interesting, first of all, to note the various animals which come more especially within the scope of the study of the veterinarian. Of course the common domesticated animals all belong to the vertebrate subkingdom, and the two classes with which he has chiefly to deal are the aves and the mammalia. As to the orders of the former—the ratitæ and the carinatæ—his attention and study are directed to each, while the orders of the mammalia class which claim his attention are (1) the ungulata, with its suborders, the perissodactyla and the artiodactyla. Included in the first of these are the horse, ass, mule, zebra, quagga, etc., and in the second, ruminantia, the ox, sheep, goat, camel, etc. Non-ruminantia are represented by the porcine tribe. (2) To the carnivora, to which belong the cat, dog, etc.

Going back to the subject of anatomy, or zoötomy, which means the anatomy of animals, let us for a moment glance at the subsections with which the student of comparative medicine has to be familiar. His first object may be to investigate exclusively the structure of a particular species; this is special anatomy. He may desire to bring under observation the structure of different species of animals, in order to trace their points of resemblance, and would then be laboring in the field of comparative anatomy. If concerned with the elementary cells and tissues of which the various organs of the body are built up, his work would be histological anatomy. And, further, if tracing up the succession of forms exhibited by the individual, from the date of conception to the period of adult life, he would be making a study of developmental or embryological anatomy. And, still further, were the student endeavoring to group together facts relating to structure, in order to discern the natural laws that determine the form of different parts of the body, he would be viewing the subject from a morphological stand-point. These three latter are subdivisions of special anatomy; but there are two distinct ways in which the special anatomy of an animal may be prosecuted. In the first of these the different groups or systems of similar organs are studied separately and in the order in which one would naturally proceed had we the power to build up an animal; this is termed systematic or descriptive anatomy. By the second method the student would investigate the different structures in the order of their position in the same part of the body, such as he would find them presented in the course of a dissection; this constitutes topographical anatomy. In the first case he would be proceeding by synthesis, in the second by analysis, for the purpose of familiarizing himself with the structure of the animal body. So much, then, for a brief allusion to structure.

Being brought daily in contact with pathological conditions, a knowledge of zoötomy alone would avail the veterinarian but little without an acquaintance with normal function. Hence, a knowledge of physiology is absolutely imperative. And, while human physiology is confined to the vital phenomena of man alone, comparative physiology treats also of the functions of animals below man, with a consideration of the means by which different functions are accompanied by different animal forms. And it is only necessary to allude to the animals included in the orders previously mentioned to give some idea of the extent of the knowledge required of the veterinarian on this subject.

But, although a familiarity with structure and function is of prime importance to the student, he would be poorly equipped did he not possess a knowledge of the various fuel-materials in the form of foods to run his machine. Foods are, of course, usually dealt with under the head of physiology; but what I desire to bring out here is that on account of the greater number of the animals with which the veterinaran has to deal belonging to the herbivora, and therefore bringing him in close touch with the vegetable world, the study of botany is essential to an intelligent understanding of the subject. I fancy there is perhaps a tendency on the part of some of our medical colleges, both human and veterinary, to disregard this subject, or, at all events, limit it to pharmaceutical botany. There are, however, some of our American schools, and I think the great majority of the European schools, that give quite an extended course, embracing both the phanerogamia and the cryptogamia, their morphology, physiology, classification, etc. This is another study in the curriculum of the veterinarian which opens up to him an interesting field as a naturalist. But, although his botanical information on leaving college may be extensive, he has, as a rule, by force of circumstances, to limit the greater part of that information to the more economic purposes for which his daily professional work calls. This would include the natural orders to which the more common food-crops belong, such as graminiæ, leguminosæ, cruciferæ, solanaceæ, etc. Besides these he ought, of course, to be familiar with the orders to which the principal vegetable drugs belong, as atropaciæ, papaveraceæ, scrofularaceæ, liliaceæ, ranunculaceæ, etc. And from his knowledge of the subject he ought to be able to recognize noxious and toxic plants which are either known or supposed to be injurious to the domestic animals. And, in addition to these, his knowledge should extend to a familiarity with the various parasitic and saprophytic vegetable fungi from which the higher orders of plants suffer, and in consequence are depreciated in nutritive value as well as sometimes rendered dangerous to the health of animals.

So far, then, I have touched briefly upon anatomy and physiology, and incidentally upon botany, but when we come to the subject of pathology the field again broadens out, since living organisms, in the form of pathogenic bacteria, as the result of patient scientific investigation, have been found to be the chief causative factors in disease. Not only, then, is it necessary for the student of comparative medicine to have a knowledge of the structure and functions of the larger and higher forms of both animal and plant life, but in this age of scientific advancement it is imperative that he be familiar also with the morphology and physiology of those minute forms which have been found to be responsible for so much destruction to both life and property.

If I might be allowed a slight digression at this point I would like to add that the close relationship existing between the work of the veterinarian along bacteriological lines and the public health is not perhaps sufficiently known to be fully appreciated, more especially in the Southern States. In sections of our own country more advanced in the knowledge of this subject, and in European countries, the veterinary profession of to-day is looked upon as an indispensable factor in the conservation of the public health, as well as that immense item of public wealth which is represented by "the cattle upon a thousand hills." It is, however, the knowledge which the modern veterinarian is required to possess relative to diseases of bacterial origin in the lower animals, and which are communicable to the human family, that I desire to emphasize; and also of the changes brought about by pathogenic bacteria in the meat-supply and milk-supply of our people, which render both inimical as articles of human food.

Included in the study of the morphology and physiology of these schizomycetes is, of course, their composition, form, movement, peculiarities of reproduction, the phenomena of respiration and nutrition, the circumstances affecting their growth, such as the nature of the soil, temperature, gases, light, etc., and a very important part of the knowledge required regarding the pathogenic forms is a familiarity with their chemical products or the products of the metabolism induced by them, which may be classified as (1) ptomaïns or alkaloids; (2) albumoses or toxalbumins; and (3) enzymes—the first two being directly poisons and the third harmless, except in the presence of proteids, which they are said to be capable of transforming into poisonous albumoses.

Pathology, so far as studying it in these microscopic death-dealers, for the purpose of revivifying and reinstating them in a condition of health and vigor, is, of course, out of the question. Our main object is their destruction, and for this purpose various methods and agents are adopted, which it is unnecessary to mention on this occasion; but I might allude, just in a word, to the fact that in many instances these very pathogenic organisms have been and are being utilized for the production of a therapeutic agent peculiar to each, by which the disease of which they are the specific cause can be prevented and often cured. These are the antitoxins and vaccines, and we are all more or less familiar with records concerning vaccination in smallpox, and serum-therapy in diphtheria, tetanus, and some other hitherto most fatal of ailments.

The word germ, in the ordinary acceptation of the term, seems always to carry with it the stigma of disease; but we must not forget that although the pathogenic forms are both plentiful and ubiquitous, to a large extent, there are immense numbers of bacteria that are harmless, and many that are really of commercial or economic value. Our plants receive their nourishment through their agency; our butter is flavored by them; the ripening of the cream to make our butter, and the ripening of our cheese, are all phenomena brought about by fermentation, the work of micro-organismal life, and so on. The study of bacteria, then, in which there is a life-work for the naturalist, is also embraced in the educational curriculum of the veterinarian; but in this, as in other branches of work, to become thoroughly informed he must specialize, and this is not convenient in every case. Bacteriology, however, is one of, if not the most important branch of medical science, and is just as valuable and necessary in its application to veterinary sanitary science as it is in its relation to health and disease among the human family.

But, besides the requirements of a familiarity with this most important group of parasites belonging to the vegetable kingdom (phytoparasites) there are three sections of the animal kingdom containing parasites (zoöparasites) of the domestic animals, some of which from a pathological stand-point may be considered of almost equal importance, for the reason that some of the members of at least one of the sections in certain stages of their life-history are not only pathogenic in the lower animals, but in the human family also. I refer to the protozoa, the entozoa, and arthropodes. Here, again, we have a study which furnishes an abundance of material for the naturalist along special lines.

When touching upon the subject of botany I made general allusion to parasitic vegetable fungi. I might here make special mention of one or two groups with which we come in contact in the study and practise of comparative pathology, viz.:

1. The dermatophytes, as the tricophyton tonsurans and the achorion schonleinii, which live on the skin; the former producing tænia tonsurans, or common ringworm, the latter favus, or honeycomb ringworm.

2. The saccharomyces, as the oidium albicans, which infest the upper portions of the digestive canal and produce what is known as parasitic stomatitis; also the saccharomyces guttulatus, sometimes found in the digestive canal of various herbivora.

3 The haplococcus reticulatus, a parasite of the muscular tissues of the hog.

4. Several kinds of aspergillus, "moulds," belonging to the perisporiacæ, which may develop in the air-passages of birds and of some mammals.

There are, of course, others, but the above will suffice, as we have not the time for more than passing reference.

Of the protozoa, only three classes contain parasites of the domesticated animals. These are the amœba, sporozoa, and infusoria. Of the first of these is the "amœba parasitica," which has been discovered in ulcers in the lips and feet of sheep. The coccidia and the sarcosporidiæ, belonging to the psorospermiæ, are the principal representatives of the sporozoa affecting the work of the veterinarian. The coccidiæ chiefly infest the digestive canal, the sarcosporidiæ being found exclusively in the muscular tissue. The infusoria found in the domestic animals, and observed more particularly in the alimentary tract or its accessories belong to the subclass flagellata or ciliata. We have also the pyrosoma bigeminum of tick-fever, which is a protozoon.

(To be continued.)

